# Patient satisfaction and efficacy of calcipotriol plus betamethasone dipropionate gel in plaque psoriasis patients with poor adherence

**DOI:** 10.1111/1346-8138.15522

**Published:** 2020-07-30

**Authors:** Hidetoshi Takahashi, Hiroyasu Katayama, Yuta Uwajima, Masato Koda, Hajime Sasaki, Katsumi Tanito, Masanori Hagiwara, Koma Matsuo, Hidemi Nakagawa

**Affiliations:** ^1^ Takagi Dermatology Clinic Obihiro Japan; ^2^ Sagamiono Skin Clinic Sagamihara Japan; ^3^ Uwajima Dermatology Clinic Tokyo Japan; ^4^ Aozora Dermatology Clinic Tokyo Japan; ^5^ Oyumino Rainbow Skin Clinic Chiba Japan; ^6^ Tsukuda River‐City Dermatology Clinic Tokyo Japan; ^7^ Toyosu Park city Skin Clinic Tokyo Japan; ^8^ Nakano Dermatology Clinic Tokyo Japan; ^9^ Atago Dermatology Clinic Tokyo Japan

**Keywords:** adherence, patient satisfaction, patient treatment preference, plaque psoriasis, topical treatment

## Abstract

Poor adherence to treatment makes achievement of expected therapeutic outcomes more difficult, especially in chronic disorders like psoriasis. There are several critical factors that affect adherence, including therapeutic efficacy, patient satisfaction, patient treatment preferences and ease of application, especially in topical therapy. The fixed combination of calcipotriol plus betamethasone dipropionate in a gel formulation (Cal/BDP gel) has been recommended as a first‐line topical treatment for mild to moderate plaque. To examine whether Cal/BDP gel can effectively improve treatment adherence, we investigated the effects of once‐daily Cal/BDP gel on factors affecting adherence at weeks 4, 8 and 12 in patients with plaque psoriasis who had poor adherence. A total of 46 subjects were enrolled and 41 subjects (26 men, 15 women; mean age, 50.5 years) were included in the analysis. The following items were evaluated: Patient Preference Questionnaire, nine‐item Treatment Satisfaction Questionnaire for Medication, Physician’s Global Assessment (PGA), modified Psoriasis Area and Severity Index (m‐PASI), body surface area (BSA), pruritus, medication adherence and application time. In patients with poor adherence, many preferred treatment with Cal/BDP gel and evaluated its convenience as “excellent” at weeks 4 and 12. At week 12, the proportion of “clear”/”very mild” ratings using PGA reached 20.5%, the change from baseline on m‐PASI was −61.3% and the change from baseline on BSA was −39.8%, suggesting that the skin symptoms of psoriasis had improved greatly. In most patients, the longer they used Cal/BDP gel, the greater their preference and satisfaction and the higher the therapeutic effect, which increased markedly over 12 weeks. These results suggest that Cal/BDP gel can effectively improve treatment adherence. Conversely, high adherence to Cal/BDP gel must enhance the therapeutic effect. Therefore, we expect that Cal/BDP gel could become the mainstay of topical psoriasis treatment in patients with poor adherence.

## INTRODUCTION

Poor adherence to treatment is a ubiquitous problem in clinical practice and may explain poor treatment outcomes, especially when medication is not well accepted by patients.^1^, ^2^ In particular, patients with chronic disorders are less likely to adhere to treatment regimens than patients with acute disorders.^3^ Psoriasis is a chronic skin disorder associated with physical and mental disability and it represents a significant public health challenge.^4^, ^5^ As with other chronic diseases, poor adherence to treatment is common among patients with psoriasis.^6^ Topical therapies play an important role and remain the mainstay of psoriasis treatment, with approximately 90% of patients in Japan using topical medications.^7^ Because the long‐term management is mandatory for patients with psoriasis, the maintenance of good adherence, especially in topical treatments, is key to achieving patient satisfaction.^8^ Poor therapeutic outcomes from topical therapies in psoriasis are often encountered due to poor adherence and ineffective use.^1^ Therefore, maintaining good adherence is a key factor affecting the real‐world effectiveness of topical treatments for chronic disorders like psoriasis.^9^ Reports indicate that poor adherence is further exacerbated by the need to apply topical drugs, as this may be troublesome and time‐consuming, especially when combined with patients’ low acceptability of certain messy treatment vehicles.^10^ Recently, the fixed combination of calcipotriol and betamethasone dipropionate in a gel formulation (Cal/BDP gel) has been recommended as a first‐line topical treatment for mild to moderate psoriasis,^2^, ^8^ and demonstrated great superiority in terms of both ease of application and non‐stickiness in Japanese patients with plaque psoriasis.^11^ Cal/BDP gel is thought to improve adherence to treatment and show a superior therapeutic effect in actual clinical settings.^11^ There are several critical factors that affect adherence, including therapeutic efficacy, patient satisfaction, patient preferences for treatment and ease of application, especially in topical therapy.^1^, ^8^ By understanding and manipulating the factors that affect treatment adherence, improvement in adherence could lead to better control and outcomes in topical psoriasis treatment.^1^ In this study, to examine whether Cal/BDP gel is useful for improving treatment adherence, we investigated the effects of Cal/BDP gel on the aforementioned factors affecting adherence in patients with plaque psoriasis on the body who had poor adherence to their previous topical treatments (<60%).

## METHODS

### Study design

This was designed as a multicenter, single‐arm, open‐label clinical study in patients with plaque psoriasis on the body, who showed poor adherence to their previous topical treatments. This study was performed in accordance with protocols approved by the Certified Review Board of the Medical Corporation Okinawa Tokushukai, the Declaration of Helsinki and the Clinical Trials Act. All patients provided written informed consent to participate in this study. The outline of this study was registered and published in the Japan Registry of Clinical Trials (trial ID no. jRCTs031180049).

### Study subjects

The major inclusion criteria were: (i) patients with plaque psoriasis on the body (trunk and/or limbs, including those with psoriatic arthritis); (ii) patients with plaque psoriasis who showed poor adherence (<60% of application of topical drugs as directed by their doctor), did not apply topical drugs as directed by their doctor and did not achieve expected clinical outcomes during 4 weeks of treatment with topical drugs other than the investigational product (topical activated vitamin D_3_, topical steroids or in‐house mixed preparations of these drugs, or topical fixed combination drugs containing topical steroid and activated vitamin D_3_) before the start of this study; (iii) patients with psoriasis affecting 30% or less of their body surface area (BSA); (iv) patients with a Physician’s Global Assessment (PGA) rating of “mild” or worse; and (v) patients aged 20 years or older who provided written, informed consent to participate in this study. In addition, the adherence rate was calculated based on the number of applications instructed by the doctor and the number of applications actually performed, which was interviews with the patient.

The exclusion criteria were: (i) women who were pregnant, possibly pregnant or wished to become pregnant during the study period; (ii) breast‐feeding women; (iii) patients with known allergy or possible allergy to any component of the investigational product; (iv) patients with bacterial, fungal, spirochetal or viral skin infection or parasite infestation, and patients with any diseases that could potentially be aggravated; (v) patients with skin ulcers and those with second‐ or third‐degree burns or frostbite; (vi) patients who had previously used the investigational products for treatment of lesions on the body; (vii) patients who had received etretinate within 6 months before the start of study treatment for men or within 1 year before the start of study treatment for women; (viii) patients who had received systemic treatment with biologics before the start of study treatment; (ix) patients with evidence of severe renal failure, liver dysfunction or cardiac disease; (x) patients with hypercalcemia; and (xi) other patients who were considered by the investigator to be unsuitable for the study.

### Treatment with the investigational product

Once daily for 12 weeks, the subjects applied an appropriate amount of the fixed combination of calcipotriene 0.005% plus betamethasone dipropionate 0.064% (Cal/BDP) gel to lesions of plaque psoriasis on the body. The maximum weekly dose allowed was 90 g.

### Assessment of efficacy and safety

The primary outcomes of this study were: (i) drug preference of the subjects at weeks 4 and 12 (determined using the Patient Preference Questionnaire [PPQ]);^12^ (iii) comparison of patient satisfaction (nine‐item Treatment Satisfaction Questionnaire for Medication [TSQM‐9])^13^, ^14^ between baseline and weeks 4 and 12; and (iii) comparison of the PGA between baseline and weeks 4 and 12. The PPQ collected data on the patient’s preference for their current treatment compared with their previous treatments. The PPQ contains five questions using a 4‐point Likert format (1, “strong agreement”; 2, “agreement”; 3, “disagreement”; and 4, “strong disagreement”) and a supplementary option “does not apply to me”.

The secondary outcomes were: (i) comparison of medication compliance rate between previous treatments and the study treatment at weeks 4, 8 and 12; (ii) comparison of the application time between previous treatments and the study treatment at weeks 4, 8 and 12; (iii) comparison of PGA between baseline and week 8; (iv) comparison of the mean modified Psoriasis Area and Severity Index (m‐PASI) and mean percent change between baseline and weeks 4, 8 and 12; (v) comparison of patient satisfaction (TSQM‐9) between baseline and week 8; (vi) comparison of the BSA affected by psoriasis between baseline and weeks 4, 8 and 12; and (vii) comparison of pruritus between baseline and weeks 4, 8 and 12 using a numerical rating scale (NRS). NRS categories were defined as 0 for “no pruritus”, 1–3 points for “mild pruritus”, 4–6 points for “moderate pruritus”, 7–8 points for “severe pruritus” and 9 or more points for “very severe pruritus”.^15^

The incidence and severity of adverse events and adverse drug reactions were examined. The adverse events and adverse drug reactions were coded using MedDRA/J version 21.0. Safety was indicated by the number and rate of adverse events and adverse drug reactions.

### Statistical analysis

All applicable statistical tests were performed using a two‐sided, 5% significance level. SAS version 9.4 (SAS Institute, Cary, NC, USA) was used to perform the statistical analyses. In the statistical analysis, drug preference (using PPQ) of the subjects at weeks 4 and 12 consisted of the point estimate and 95% confidence interval (CI) of the percentage of subjects who achieved “agreement” or better were calculated for each item at each time point. Comparison of patient satisfaction (TSQM‐9) between baseline and weeks 4, 8 and 12 was analyzed using the Wilcoxon signed‐rank test. Comparison of PGA between baseline and weeks 4 and 12 comprised the point estimate and 95% CI of the percentage of subjects with PGA ratings of “mild” or lower was calculated at each time point, and comparison of PGA ratings between baseline and weeks 4, 8 and 12 was analyzed using the Wilcoxon signed‐rank test. Comparison of the medication compliance rate between previous treatments and study treatment at weeks 4, 8 and 12 was analyzed using the Wilcoxon signed‐rank test. Comparison of the application times between previous treatments and the study treatment at weeks 4, 8 and 12 was analyzed using the Wilcoxon signed‐rank test. Comparison of the mean m‐PASI and mean percent change between baseline and weeks 4, 8 and 12 was analyzed using the paired *t*‐test. Comparison of the mean BSA and mean percent change between baseline and weeks 4, 8 and 12 was analyzed using the paired *t*‐test. Comparison of pruritus between baseline and weeks 4, 8 and 12 was analyzed using paired *t*‐tests.

## RESULTS

### Patient profile

**Figure 1:**
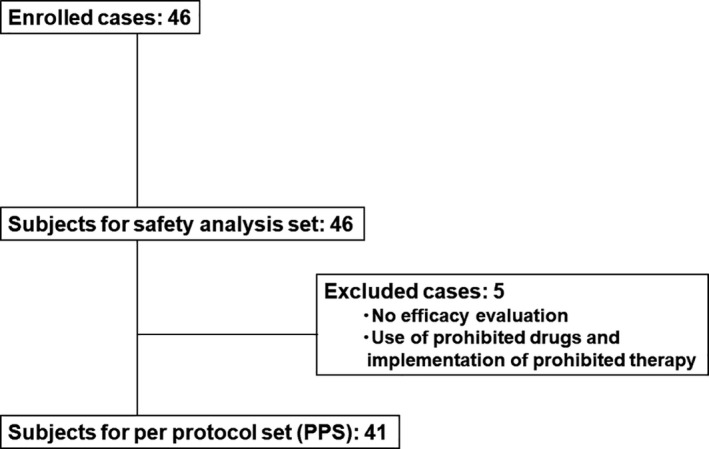
Patient disposition.

A total of 46 Japanese subjects were registered at nine dermatology clinics and constituted the safety analysis set. However, five patients were excluded from the analyses due to violation of the treatment protocol. The remaining 41 patients completed the study and constituted the per protocol set (PPS) (Fig. 1).

The patient characteristics in the PPS, as shown in Table 1, are summarized as follows. The study population comprised 26 men (63.4%) and 15 women (36.6%) with a mean age (±standard deviation [SD]) of 50.5 ± 15.6 years. Baseline PGA scores were “mild” in 15 patients (36.6%) and “moderate” in 26 patients (63.4%) (Fig. 4). The mean (±SD) m‐PASI score at baseline was 5.05 ± 3.04 (Table 2). The mean (±SD) evaluation of pruritus using the NRS was 3.7 ± 2.2.

**Table 1:** Patient characteristics

	Safety analysis set (*n* = 46)	PPS (*n* = 41)
Sex		
Male	30 (65.2%)	26 (63.4%)
Female	16 (34.8%)	15 (36.6%)
Age (years)		
Mean ± SD	50.9 ± 15.0	50.5 ± 15.6
Median	50	49
Range	26–94	26–94
Height (cm)		
Mean ± SD	163.1 ± 8.3	162.3 ± 8.2
Median	163	160
Range	143–180	143–180
Weight (kg)		
Mean ± SD	65.1 ± 13.2	63.6 ± 13.0
Median	63	62
Range	40–95	40–95
Duration of psoriasis (years)		
Mean ± SD	14.7 ± 12.4	13.4 ± 11.4
Median	10	9.5
Range	0.6–41.0	0.6–41.0

Data are expressed as number (%). PPS, per protocol set; SD, standard deviation.

**Table 2:** The mean score for m‐PASI and change from baseline

	Score	*P*‐value for difference	Change from baseline (%)	*P*‐value for percent change
Baseline	5.1 ± 3.0		‐	
Week 4	3.9 ± 3.0	0.009	−18.8 ± 49.7	0.020
Week 8	2.4 ± 1.6	<0.001	−47.7 ± 29.4	<0.001
Week 12	2.0 ± 1.8	<0.001	−61.3 ± 24.3	<0.001

Data are expressed as mean ± SD.

### Patients’ preference

**Figure 2:**
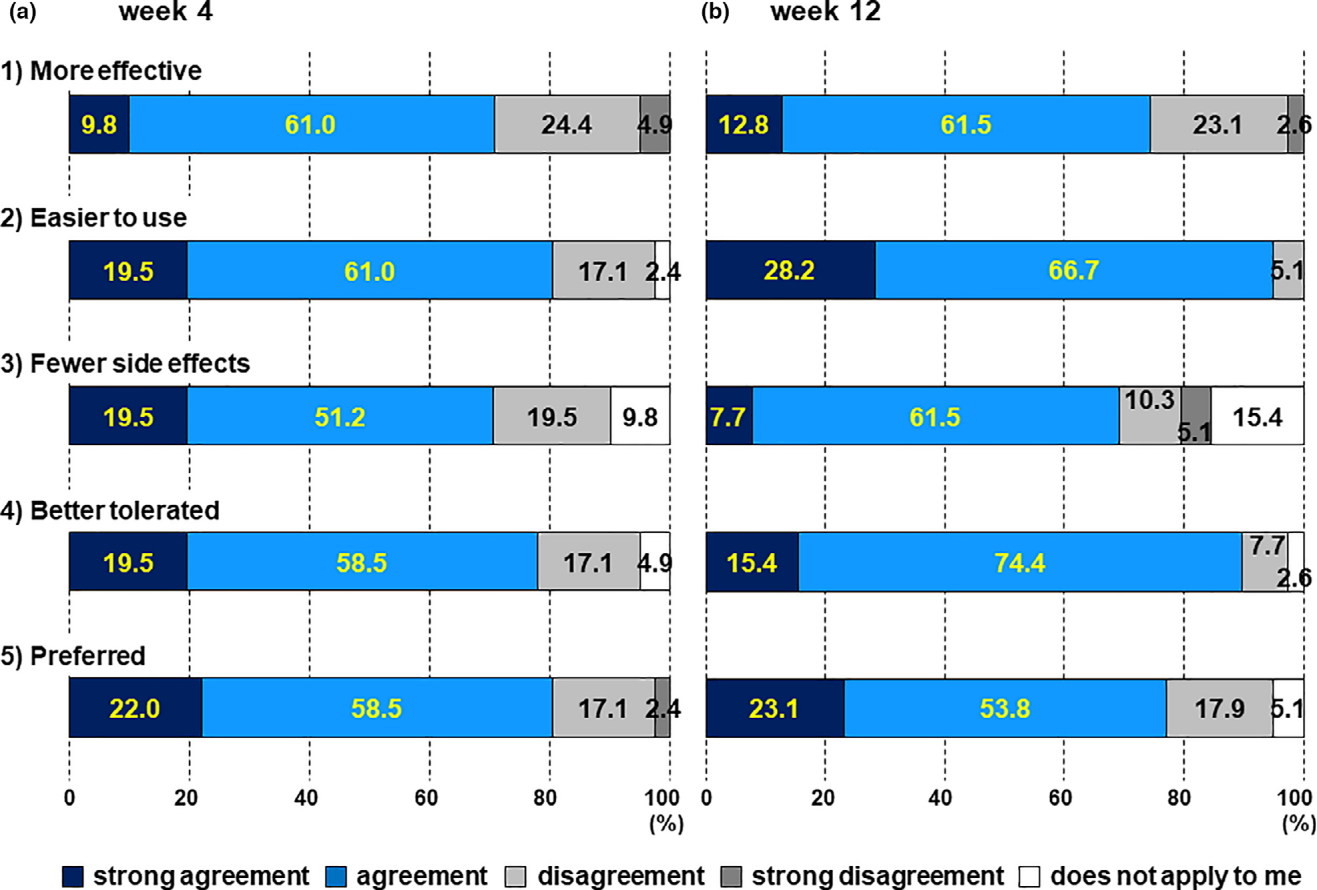
Patient preference questionnaire (PPQ) at weeks 4 and 12. PPQ at weeks (a) 4 and (b) 12. The bars respectively represent: (1) “more effective”, (2) “easier to use”, (3) “fewer side‐effects”, (4) “better tolerated” and (5) “preferred”. The numbers in the boxes indicate the percentage of answers for each option.

Patients’ preference for the current treatment with Cal/BDP gel compared with their previous treatments was assessed using the PPQ at weeks 4 and 12. In all five questions, the current treatment with Cal/BDP gel was highly preferred over the previous topical treatments (Fig. 2). More specifically, many patients rated the current topical treatment with Cal/BDP gel as being “more effective”, “easier to use”, having “fewer side‐effects”, “better tolerated” and “preferred” compared with their previous treatments at both weeks 4 and 12 (Fig. 2).

In particular, the vast majority of patients stated “strong agreement” or “agreement” at week 12 in response to the two questions about the treatment being “easier to use” and “better tolerated”. The percentage of “strong agreement” and “agreement” for both questions was discernibly higher at week 12 than at week 4 (“easier to use”, 80.5% and 94.9%; “better tolerated”, 78.0% and 89.8%, at weeks 4 and 12, respectively) (Fig. 2).

### Treatment satisfaction

**Figure 3:**
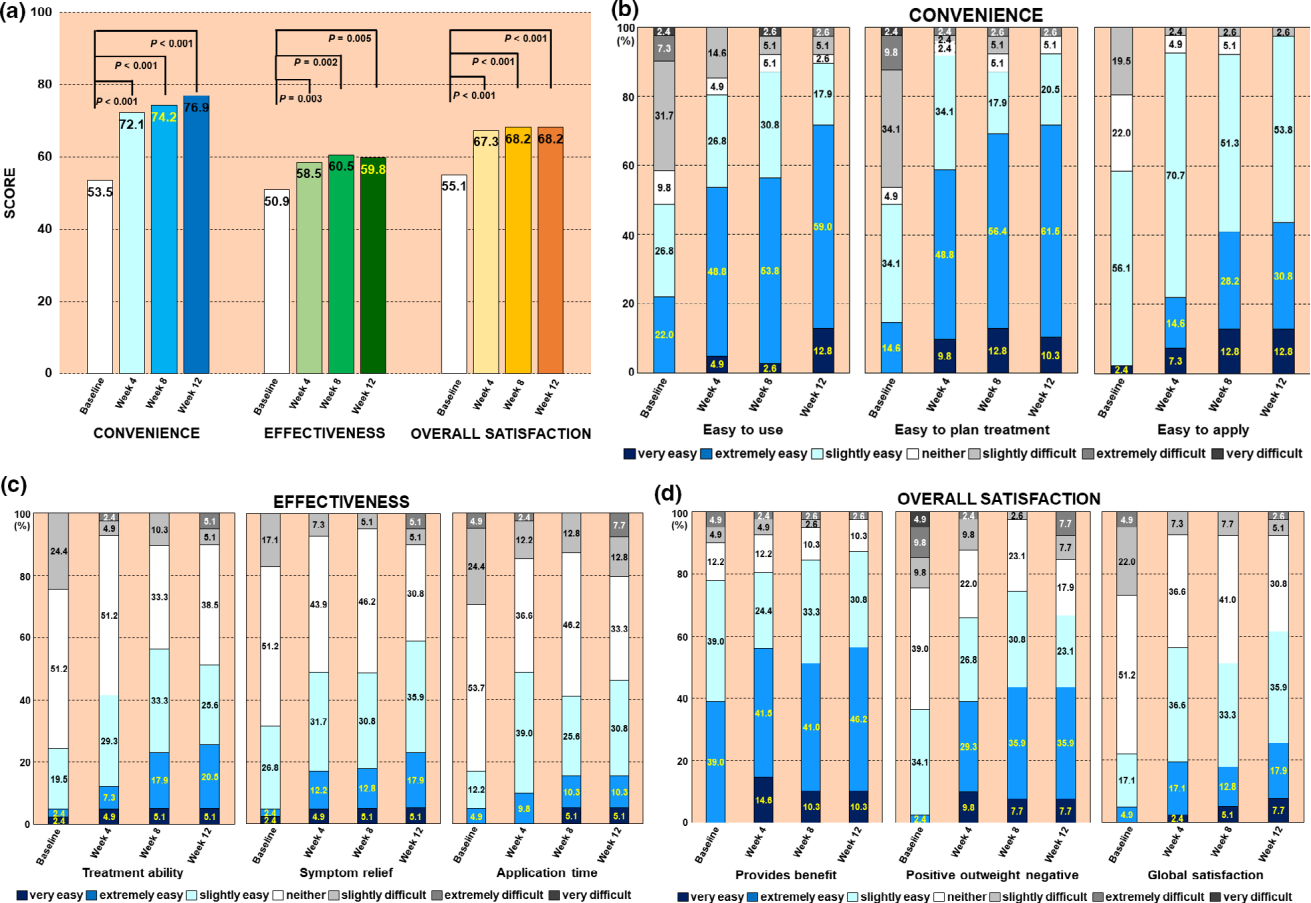
Patient satisfaction using nine‐item Treatment Satisfaction Questionnaire of Medication (TSQM‐9) at baseline and weeks 4, 8 and 12. (a) The time‐dependent changes in the three large categories for mean TSQM‐9 score are shown. The numbers in the bars indicate the mean TSQM‐9 scores for the various answers. (b) Detail: items for “convenience”. (c) Detail: items for “effectiveness”. (d) Detail: items for “overall satisfaction”. The numbers in the boxes indicate the percentage for each answer.

Treatment satisfaction was assessed using the TSQM‐9 (comprising “convenience”, “effectiveness” and “overall satisfaction” domains) at baseline and weeks 4, 8 and 12. As shown in Figure 3(a), patients with poor adherence were significantly more satisfied with Cal/BDP gel treatment than at baseline (pretreatment), at all evaluated points. Particularly concerning “convenience”, the longer the Cal/BDP gel was used, the more the convenience score tended to increase (Fig. 3a). Remarkable satisfaction was obtained for each of the individual items in “convenience”, namely “easy to use”, “easy to plan treatment” and “easy to apply” (a statistically significant difference [*P* < 0.001] from baseline for all evaluated points and all items) (Fig. 3b). Additionally, the rates of “very easy” and “extremely easy” scores for the three individual items increased after the switch to Cal/BDP gel and continued to increase until week 12 (Fig. 3b). In more detail, the combined percentage of “very easy” and “extremely easy” in “easy to use” was 22.0% at baseline and, after switching to Cal/BDP gel, increased over time to 53.7% and 56.4% at weeks 4 and 8, respectively. Finally, this measure reached 71.8% in week 12. Regarding the “easy to plan treatment” item, the proportions of “very easy” and “extremely easy” were 14.6%, 58.6%, 69.2% and 71.8% at baseline and weeks 4, 8 and 12, respectively. The proportion of “very easy” and “extremely easy” for the “easy to apply” item was 2.4% at baseline and, after switching to Cal/BDP gel, increased to 21.9% and 41.0% at weeks 4 and 8, respectively. This measure reached 43.6% at week 12 (Fig. 3b).

The proportions of “very satisfied” and “extremely satisfied” in the individual items of “effectiveness” also tended to increase over time after switching to Cal/BDP gel and increased at each evaluated point with significant differences from baseline (Fig. 3c). The proportions of “very satisfied” and “extremely satisfied” for the individual items of “overall satisfaction” significantly increased in the 4 weeks after switch to Cal/BDP gel, and then plateaued until week 12 (Fig. 3d).

### Efficacy evaluation

#### Objective evaluation by physicians

**Figure 4:**
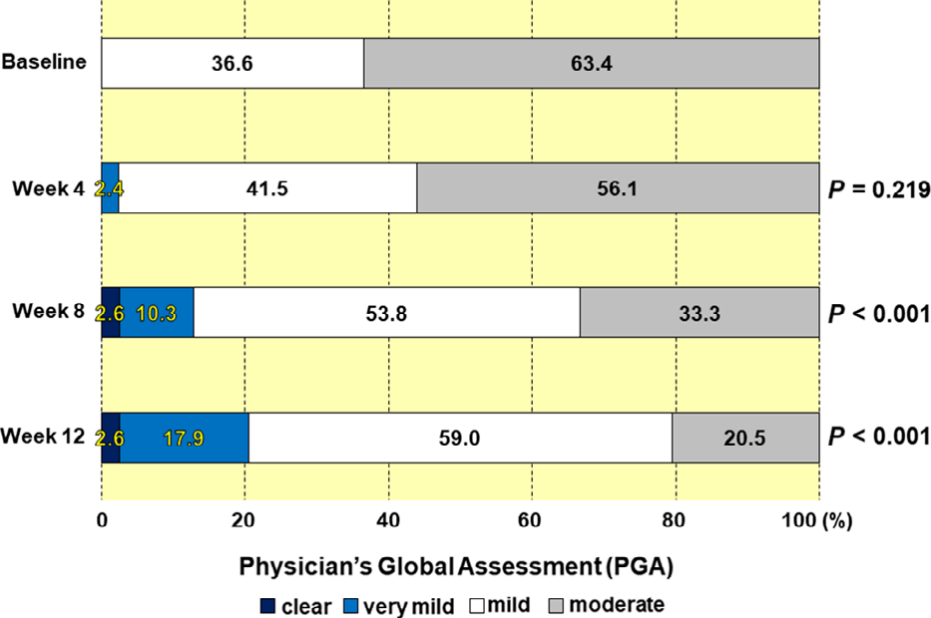
Effectiveness evaluation using Physician’s Global Assessment at baseline and weeks 4, 8 and 12. The numbers in the boxes indicate the percentages in the various categories.

Evaluation of efficacy at weeks 4, 8 and 12 was conducted using PGA, m‐PASI and BSA. The combined percentages of “clear” and “very mild” as evaluated by PGA were 0.0%, 2.4%, 12.9% and 20.5% at baseline and weeks 4, 8 and 12, respectively (Fig. 4). The rates of change in m‐PASI from baseline were −18.8%, −47.7% and −61.3% at weeks 4, 8 and 12, respectively (Table 2). The rates of change in BSA from baseline were −11.1%, −28.1% and −39.8% at weeks 4, 8 and 12, respectively (Table 3). In those patients with poor adherence, the therapeutic effects of Cal/BDP gel as assessed by PGA, m‐PASI and BSA all increased until week 12. Specifically, at week 12, the proportion of “clear” and “very mild” as assessed by PGA reached 20.5% (Fig. 4), the rate of change in m‐PASI from baseline was −61.3% (Table 2) and the rate of change in BSA from baseline was −39.8% (Table 3). Symptoms of psoriasis had improved greatly.

**Table 3:** Mean score for body surface area and change from baseline

	Score	*P* for difference	Change from baseline (%)	*P* for percent change
Baseline	6.2 ± 5.6		–	
Week 4	5.6 ± 5.3	0.060	−11.1 ± 26.8	0.012
Week 8	4.7 ± 5.0	<0.001	−28.1 ± 24.4	<0.001
Week 12	4.1 ± 4.9	<0.001	−39.8 ± 26.2	<0.001

Data are expressed as mean ± standard deviation.

#### Subjective evaluation by patients

**Figure 5:**
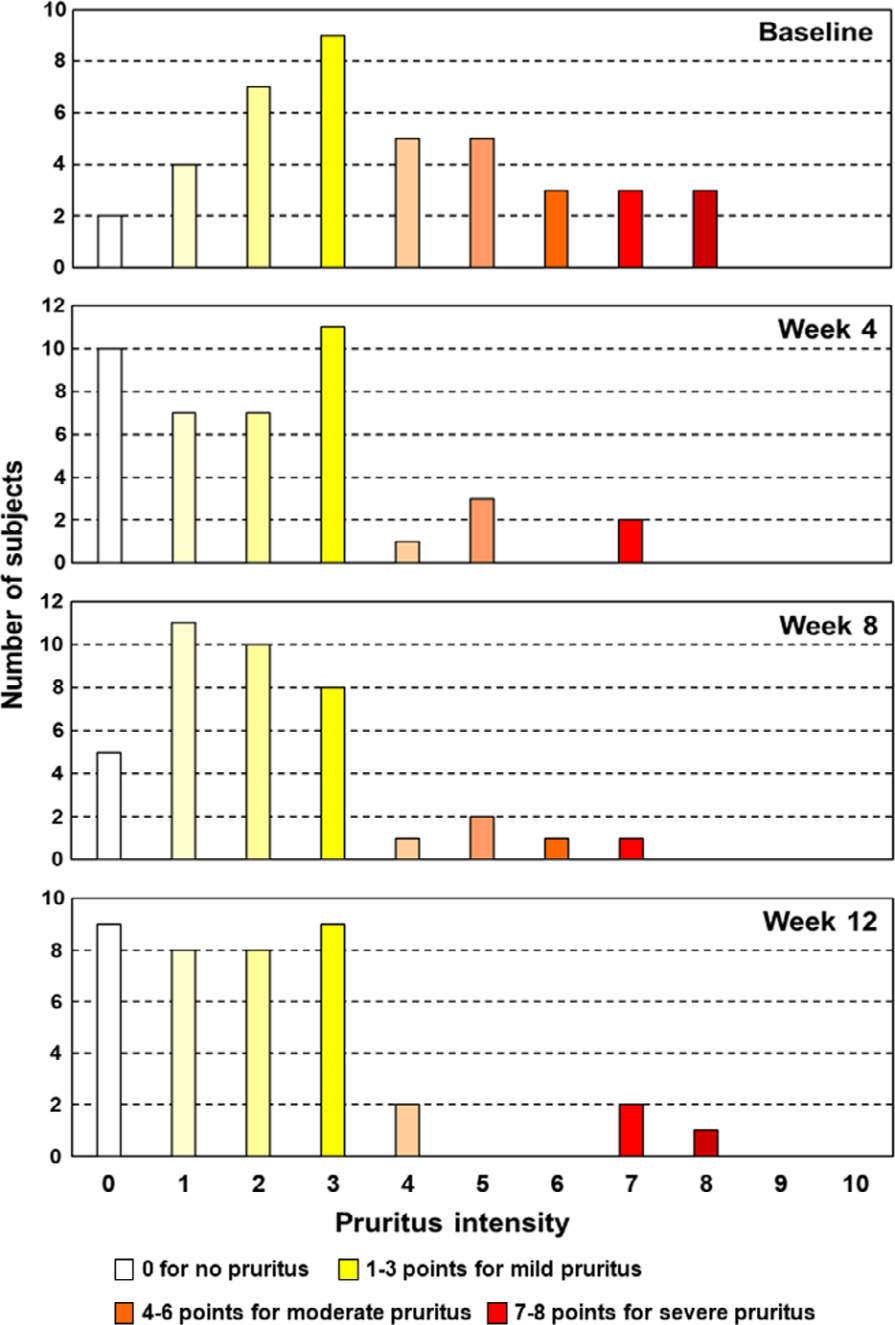
Histogram of pruritus intensity. The horizontal axis shows the score for pruritus intensity, evaluated using a numerical rating scale (NRS). The vertical axis represents the number of subjects showing each score. Based on the definition of NRS categories by Reich *et al*.,^15^ the pruritus intensity is color‐coded as: 0 for no pruritus (open), 1–3 points for mild pruritus (yellowish), 4–6 points for moderate pruritus (orangish), 7–8 points for severe pruritus (reddish) and 9 or more points for very severe pruritus.

Different from the PGA and m‐PASI, which were assessed by the physician, the occurrence of moderate to severe pruritus in the patient‐reported pruritus intensity (NRS score, ≥4)^15^ reduced from week 4 after switching to Cal/BDP gel (Fig. 5). In addition, the rates of reduction at weeks 4, 8 and 12 were 24.6%, 26.1% and 28.7%, respectively (a statistically significant difference [*P* < 0.001] from baseline at all evaluated points, data not shown). This suggests that patients could notice the beneficial effects of Cal/BDP gel soon after switching therapy.

**Figure 6:**
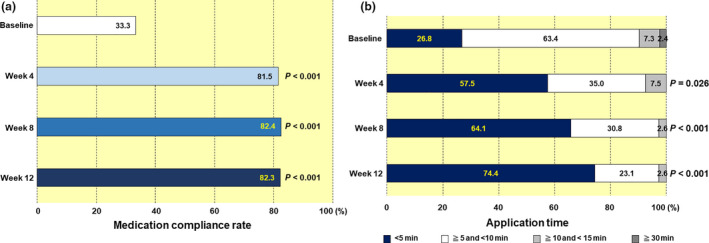
(a) Medication compliance rate and (b) application time. There was no patient with an application time of 15 min or more and less than 30 min during the study period. The numbers in the boxes indicate the percentages in the various categories.

Patients participating in this study originally had poor adherence to topical treatment, and at the start of the study, their adherence rate for topical treatment was approximately 30%. However, during the use of Cal/BDP gel, the average medication compliance rate did not decrease, with a rate that was maintained at 80% or more (Fig. 6a). In addition, by changing the previous topical treatments to Cal/BDP gel, the application time of the therapeutic agent was dramatically shortened (Fig. 6b). The proportion of patients with an application time of under 5 min was 26.8% with the previous treatments (at baseline), but after switching to Cal/BDP gel, increased over time to 57.5% and 64.1% at weeks 4 and 8, respectively. Finally, this proportion reached 74.4% at week 12 (Fig. 6b).

### Safety

In total, 12 adverse events were reported during this study. These adverse events occurred in 10 of 46 enrolled patients, with no serious adverse events (Table 4). Of these adverse events, four were causally related to the Cal/BDP gel and reported as adverse drug reactions (Table 4). The following adverse drug reactions occurred: cellulitis (one case), contact dermatitis (one case) and psoriasis (two cases). No new or serious adverse drug reactions were reported.

**Table 4:** Adverse events and adverse drug reactions

Adverse events	
No. of cases	10 (21.7%)
No. of occurrences	12
Serious adverse events	
No. of cases	0 (0.0%)
No. of occurrences	0
Adverse drug reactions	
No. of cases	4 (8.7%)
No. of occurrences	4
Serious adverse drug reactions	
No. of cases	0 (0.0%)
No. of occurrences	0

Data are expressed as *n* (%).

## DISCUSSION

Poor adherence can lead to negative effects on the effective management of psoriasis. As a result of poor adherence, the prognosis is poor and consequently medical costs have risen significantly.^16^ Improving adherence will benefit the patient and will likely result in better control of psoriasis and improved outcomes with topical treatments.^1^ In this study, to investigate whether Cal/BDP gel is useful for improving treatment adherence, we evaluated both patient satisfaction with Cal/BDP gel and its efficacy in plaque psoriasis, in patients with poor adherence with their previous topical treatments.

It has been reported that many patients with psoriasis vulgaris prefer their current treatment with Cal/BDP gel at week 8 over previous treatments.^12^ Our results showed that, even in patients with poor adherence, many patients preferred the current treatment with Cal/BDP gel compared with the treatments they had received before participating in this study. In particular, patient preference was very high with respect to two items, “easier to use” and “better tolerated”. The percentage of “strong agreement” and “agreement” was higher at week 12 than at week 4 for both items. Notably, these findings suggest that patients with poor adherence will realize these two benefits progressively and to a greater degree.

Over a 52‐week treatment period, Cal/BDP gel has been reported to provide high patient satisfaction in the PRO‐Long real‐world study (a multicenter, prospective, observational, 52‐week study).^14^ Our study also showed that, even in patients with poor adherence, treatment satisfaction with Cal/BDP gel was significantly higher (*P* < 0.001), especially regarding convenience (*P* < 0.001) and increased with the period of use of Cal/BDP gel. Based on these findings, the convenience of Cal/BDP gel is highly rated by patients with poor adherence and will likely improve adherence. In particular, “easy to plan treatment” (“How easy or difficult is it to plan when you will use the medication daily?”) and “easy to apply” (“How easy or difficult is it to take the medication as instructed by your doctor or pharmacist?”) are two questions directly related to adherence, and the high patient ratings here must correlate with improved treatment adherence. Consequently, it is clear from the results of the PPQ and TSQM‐9, evaluations of the patient’s perspective, that the convenience of Cal/BDP gel is very beneficial in patients with poor adherence, and that user‐friendliness improves treatment adherence.

In this study, even in patients with poor adherence, the therapeutic effect of Cal/BDP gel persisted in all indications, including PGA, m‐PASI and BSA, at least until week 12. In addition, from the viewpoint of the mechanism of action of Cal/BDP, the normalization of the imbalance between regulatory and pro‐inflammatory T cells by Cal/BDP is shown to contribute to the successful long‐term control of psoriasis.^17^ Therefore, it is conceivable that this sustained therapeutic effect is a major motivator for continuing long‐term treatment. Conversely, a high level of adherence to treatment due to the convenience of Cal/BDP gel will also improve its therapeutic effect.

Unlike PGA and m‐PASI, the patients’ subjective pruritus was already significantly reduced by week 4 (*P* < 0.001), suggesting that the patient can notice the beneficial effect immediately after switching to Cal/BDP gel. Patient awareness of early effectiveness, including the reduction of pruritus and other subjective symptoms, is believed to contribute to improved adherence.

Elegant cosmetic products such as gels and creams are generally considered less sticky and take less time to apply than other formulations.^2^, ^10^ A 52‐week real‐world study showed that most patients who applied Cal/BDP gel daily took 5 min or less at 52 weeks.^14^ In this study, the medication compliance rate was maintained at a high average of approximately 82% throughout the implementation period. Under these conditions, it was found that even in patients with poor adherence, application time was dramatically reduced after switching to Cal/BDP gel. The proportion of patients with an application time of under 5 min was 26.8% before switching (at baseline), but after switching to Cal/BDP gel, this increased over time to 57.5% and 64.1% at weeks 4 and 8, respectively, finally attaining 74.4% at week 12. The results of the PPQ and TSQM‐9 patient‐based evaluation indicate that approximately 90% of patients evaluated “excellent convenience of Cal/BDP gel” highly at week 12. Such patient satisfaction for Cal/BDP gel is considered to play a key role in shortening the application time. However, these facts alone cannot explain the tendency of the application time to decrease over time. We estimate that the excellent effect of Cal/BDP gel on treating body psoriasis is attributed to improving adherence. It is considered that early improvement in the patient’s symptoms including pruritus, and the subsequent decrease in BSA (lesion area, i.e. the application area) over time contributed to the shortening of application time.

This study was designed to gain insight into patient satisfaction and effectiveness in clinical practice. However, the study has the following limitations. The study was conducted in a daily practice setting, not under the strictly controlled conditions of clinical study, and with a relatively small sample of 41 patients in the PPS. Also, the observation period was relatively short (for 12 weeks) for judging the sustained therapeutic effect of Cal/BDP gel.

We evaluated treatment satisfaction and efficacy of Cal/BDP gel in patients with poor adherence to their previous topical treatments for 12 weeks. In most patients, the longer they used the Cal/BDP gel, the more its reported convenience and therapeutic effect increased over 12 weeks. Both early improvements in patients’ symptoms and a subsequent dramatic reduction in application time were demonstrated. Consequently, because of both the high level of patient satisfaction with Cal/BDP gel, especially with regard to its convenience, and its sustained therapeutic effect, it is effective in improving treatment adherence. Conversely, the high adherence to Cal/BDP gel is likely to increase its therapeutic effect. Based on these findings, we expect that Cal/BDP gel may become the mainstay of topical psoriasis treatment for patients with poor adherence.

## CONFLICT OF INTEREST

This research was funded by LEO Pharma. H. T. has received speaking fees from AbbVie and Eli Lilly Japan. K. M. has received consulting fees and speaking fees from Maruho. H. N. received consulting fees and/or speaker honoraria from AbbVie, Janssen Pharmaceutical, Japan Tobacco, Kyowa Kirin, LEO Pharma, Maruho, Torii Pharmaceutical and UCB Japan.
